# Adolescents’ mental health problems increase after parental divorce, not before, and persist until adulthood: a longitudinal TRAILS study

**DOI:** 10.1007/s00787-020-01715-0

**Published:** 2021-02-10

**Authors:** Janne M. Tullius, Marlou L. A. De Kroon, Josué Almansa, Sijmen A. Reijneveld

**Affiliations:** Department of Health Sciences, Community and Occupational Medicine, University Medical Center Groningen, University of Groningen, PO Box 196, 9700 AD Groningen, The Netherlands

**Keywords:** Parental divorce, Adolescents, Mental health problems, Occurrence of EBP, TRAILS cohort

## Abstract

Parental divorce is one of the most stressful life events for youth and is often associated with (long-lasting) emotional and behavioral problems (EBP). However, not much is known about the timing of the emergence of these EBP in adolescents relative to the moment of parental divorce, and its longitudinal effects. We therefore assessed this timing of EBP in adolescents of divorce and its longitudinal effects. We used the first four waves of the TRacking Adolescent’s Individual Lives Survey (TRAILS) cohort, which included 2230 10–12 years olds at baseline. EBP were measured through the Youth Self-Report (YSR), as internalizing and externalizing problems. We applied multilevel analysis to assess the effect of divorce on EBP. The levels of both internalizing and externalizing problems were significantly higher in the period after parental divorce (*β* = 0.03, and 0.03, respectively; *p* < 0.05), but not in the period before divorce, with a persistent and increasing effect over the follow-up periods compared to adolescents not experiencing divorce. Adolescents tend to develop more EBP in the period after parental divorce, not before. These effects are long-lasting and underline the need for better care for children with divorcing parents.

## Introduction

Between 1960 and 2016, the crude divorce rate in the European Union has risen from 0.8 to 1.9 per 1000 individuals a year making it one of the most frequent and common stressful events during one’s lifetime [1, 2]. It is estimated that more than 10 million minors in Europe are affected by parental divorce [3]. Parental divorce, both separation and legally administered divorce, has been found to increase the risk of psychological distress and depression and behavioral problems [4] in children and adolescents, making it a pressing public health challenge.

Adolescents whose parents have divorced have an increased risk of several emotional and behavioral problems (EBP), but evidence on the timing of this association is lacking. These EBP regard, e.g., acting out and social problems [4–7], a higher numbers of depressive episodes, and more difficulties adjusting to adversities and distress. Knowledge on the moment of occurrence of EBP in adolescents of divorce is limited. More specifically, it is not known if the problems occur before or after the actual parental divorce. Amato showed that the adolescent adjusts more easily if the post-divorce standard of living does not exceptionally decline and the parents maintain a positive co-parenting relationship [8]. This suggests a strong effect of post-divorce circumstances in the development of EBP. Other studies claim that increased pre-divorce family instability determines the poor EBP outcomes in children and adolescents [9], implying that EBP precede the divorce.

Additionally, a number of studies reported that a divorce at older child age is associated with less severe problems than divorce at a younger age [10]. Similarly, gender is thought to be a modifying factor as the literature reports that female adolescents are more prone to develop depressive symptoms and over-controlled behaviors than their male peers after experiencing a divorce [1, 11]. In contrast, male adolescents who have experienced parental divorce tend to be more susceptible to develop behavioral problems [1, 4, 12–15]. However, evidence lacks on the effects of age and gender on the comparison between the period before and after the parental divorce. Such evidence may help to more effectively identify the right moment to offer targeted (gender- and age-specific) interventions to adolescents of divorce.

Therefore, the aim of the current study is to assess (a) if EBP of adolescents after parental divorce persist over time, and (b) if these EBP of adolescents occur before parental divorce or after parental divorce.

## Materials and methods

### Setting and population

This study was a prospective (longitudinal) cohort study of the ‘TRacking Adolescent’s Individual Lives Survey’ (TRAILS), a large cohort of adolescents living in the North of The Netherlands. The data for this study were derived from the first four measurement waves (T1–T4) of the TRAILS sample with bi- or triennial measurements since 2001 (T1 in 2001/2002, T2 in 2003/2004, T3 in 2006/2007, and T4 in 2009/2010); thus, adolescents were followed from the ages 10–12 until 21–23. Participants from three provinces in the North of The Netherlands were recruited by approaching 135 primary schools in the provinces Groningen, Friesland, and Drenthe, of which 122 (90.4%) were willing to participate. The baseline sample included 2230 children aged 10–12-years (response rate 76%) with a mean age of 11.1 (SD 0.55), and a female participant proportion of 50.8%. Of these, for 349 participants, there was no information at any next wave, leaving an effective sample size of *n* = 1881. The TRAILS sample is highly representative of the general population comprising participants of predominantly Dutch ethnicity (89.7%) as well as diverse socioeconomic and educational backgrounds. Further information concerning the sampling procedure and the sample’s representativeness are reported elsewhere [16, 17]. Approval by the Central Committee on Research Involving Human Subjects (Dutch CCMO) was obtained prior to the beginning of the TRAILS study, and repeatedly for each wave.

### Measures

#### Emotional and behavioral problems (EBP)

The outcome variables of interest were EBP (externalizing and internalizing problem scales), measured at T1–T4, assessed through the Youth Self-Report (YSR). The YSR is a frequently used questionnaire with excellent psychometric properties to measure emotional and behavioral problems and social competencies of children and adolescents [18]. At T4, the ASR (Adult Self-Report) was used. Outcome variable scores were analyzed as continuous scores enabling to estimate smaller effects and effect changes over time.

#### Parental divorce

At each measurement (T1–T4), the occurrence of a parental divorce, meaning both separation and legally administered divorce, in the preceding period was assessed. If divorce had occurred at the period preceding measurement, then the preceding period was labeled as pre-divorce period, and the period after that measurement as post-divorce period. For example, if there had been a parental divorce between T1 and T2, the individual had a post-divorce status at T2, and a pre-divorce status at T1.

Having experienced parental divorce at T1 refers to about 11 years, i.e., from birth until age 10/11. For the other waves, this regards 2–3 years.

#### Effect modifiers

As a possible effect modifier, we considered gender.

### Statistical analyses

First, we described the background characteristics of the TRAILS population and the EBP outcome variables by wave. Second, we assessed with multilevel analysis if the trend of EBP was affected by the moment of parental divorce by creating a new set of indicator variables in order to measure the (long-term) effects of pre- and post-divorce status: a post-divorce status (i.e., being ‘yes’ in the first wave that divorce was observed) and lag variables that numbered the measurements after this post-divorce variable. The pre-divorce indicator variable had value ‘yes’ in the immediate wave previously of the observed divorce. The lag variables allow to estimate a time-varying long-term effect of the post-divorce status over EBP, depending on how far each measurement is from the divorce occurrence. The multilevel models included a random intercept, time was modeled as categorical (thus, unspecified trend shape over time), and the estimation method was maximum likelihood.

Two multilevel models were built for each outcome variable (externalizing and internalizing problems): (1) the effect of post-divorce status and of lag-variables, including gender as potential effect modifier, and (2) the previous model including pre-divorce status (but excluding the last wave (T4), as it cannot be known if a divorce has taken place after the last measurement).

Prior to all analyses homogeneity of variances and normality of the distribution of residuals were checked. All participants (*N* = 2230) were included in the models, and the statistical models use all their available information. Missing information (and loss-to-follow-up) was assumed missing at random. All analyses were carried out using IBM SPSS Version 25.

## Results

### Background characteristics

Background characteristics of the TRAILS population are shown in Table 1. The baseline sample (*N* = 2230) consisted of 50.8% girls and 49.2% boys. The mean ages at the waves T1–T4 were, respectively, 11.1 (SD 0.55), 13.6 (SD 0.53), 16.1 (SD 0.73), and 19.1 (SD 0.60) years of age. The majority of the population was identified to be in the 50% medium SES group. At baseline, 21.4% of the adolescent’s parents have already been divorced, and by T4, 31.0% of all children had experienced their parent’s divorce.

**Table 1:** Background characteristics of the TRAILS population^a^

	*N*	%	
Gender	2230	–	–
Male	1097	49.2	–
Female	1133	50.8	–
Parental SES	2188		–
25% lowest	553	25.3	–
50% medium	1084	49.5	–
25% highest	551	25.2	–
Post-divorce status T1^b^ (yes)	477	21.4	–
Post-divorce status T2^b^ (yes)	78	3.4	–
Post-divorce status T3^b^ (yes)	64	3.3	–
Post-divorce status T4^b^ (yes)	53	2.7	–

	*N*	Mean (S*D*)	Range
Age T1^b^ (in years)	2230	11.1 (0.55)	10.0–12.6
Familial vulnerability externalizing problems T1^b^	2165	0.14 (0.42)	0.0–4.3
Familial vulnerability Internalizing problems T1^b^	2159	0.55 (0.79)	0.0–3.9
Age T2 (in years)^b^	2149	13.6 (0.53)	12.2–15.2
Age T3 (in years)^b^	1819	16.1 (0.73)	14.7–18.7
Age T4 (in years)^b^	1881	19.1 (0.60)	17.9–20.9

^a^This is a cohort consisting of adolescents from the north of the Netherlands

^b^T1 in 2001/2002, T2 in 2003/2004, T3 in 2006/2007, T4 in 2009/2010

*N* sample size, *SD* standard deviation, *SES* socioeconomic status (categorized in three groups), *Post-divorce status T1-4* parental divorce during period since previous measurement, *Familial vulnerability externalizing/internalizing problems* parental history of psychopathology measured at T1 through TRAILS family history interview (FIH)

### Timing of the increase of EBP relative to the moment of parental divorce and longitudinal trends

**Fig. 1:**
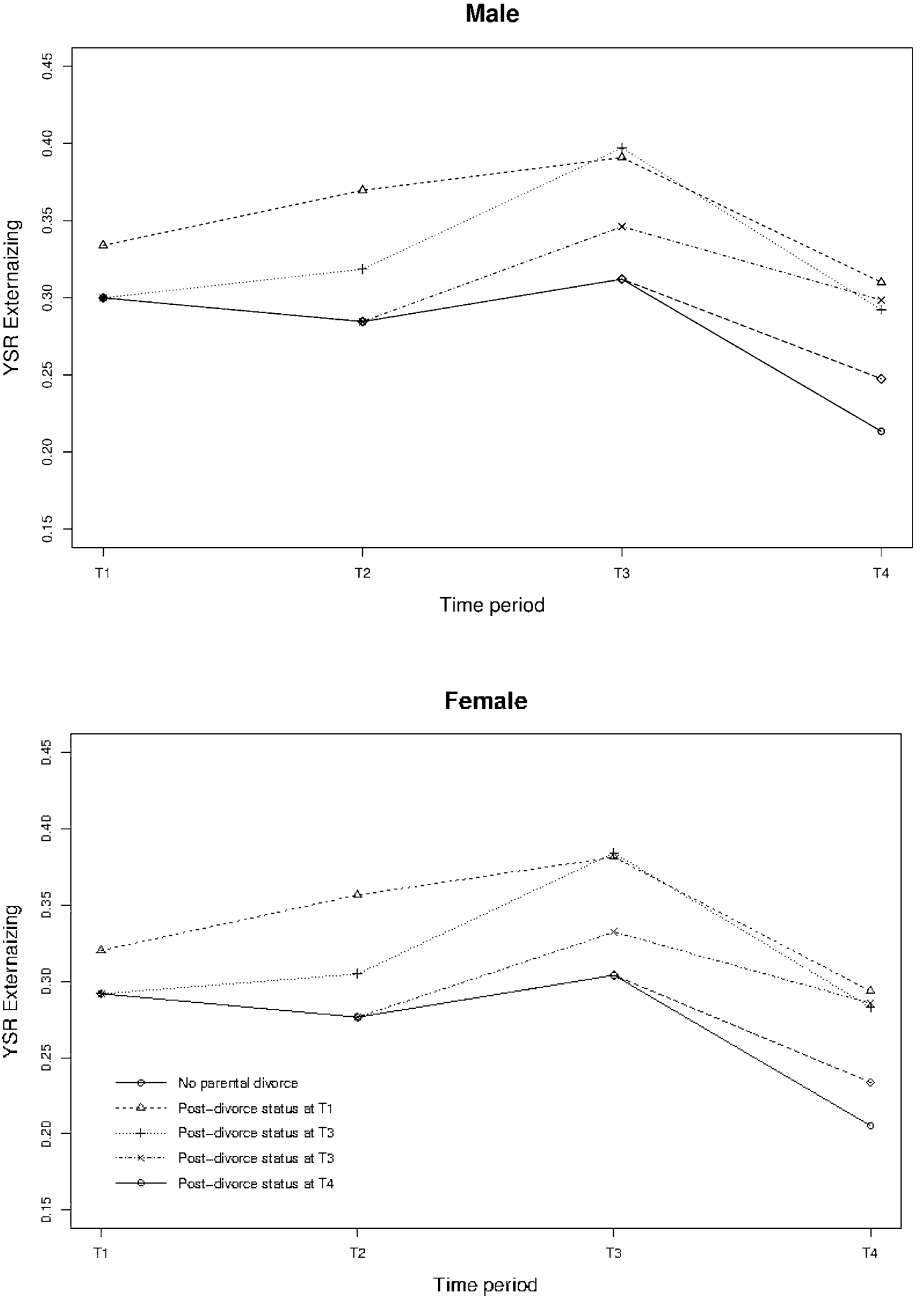

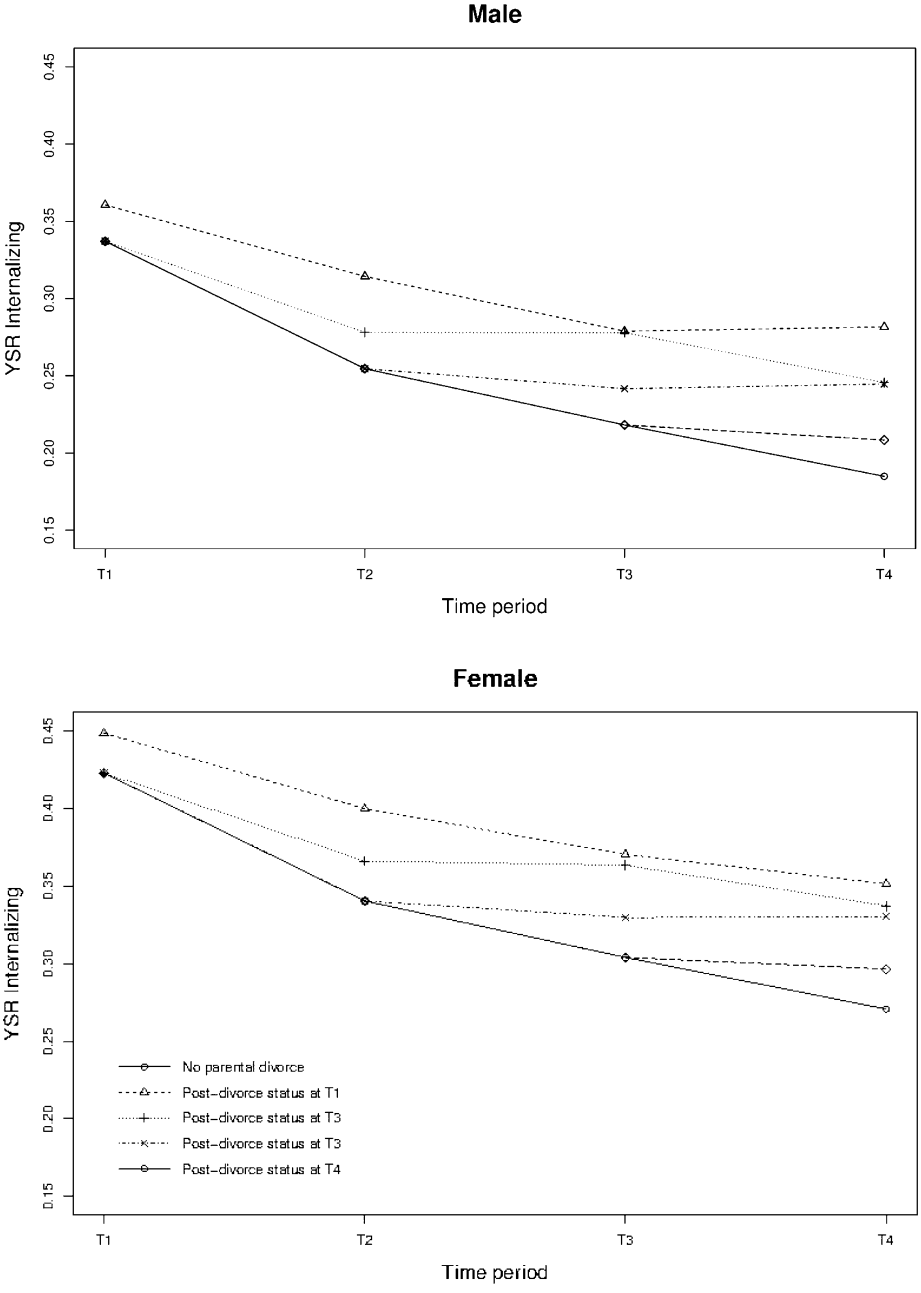
Longitudinal effects of parental post-divorce status on externalizing and internalizing problems for males and females

Table 2 shows that parental divorce resulted in a significant increase of both externalizing and internalizing problems, with a persistent and increasing effect over the following periods compared to those without divorce. Both externalizing and internalizing problems increased at the first measurement after a parental divorce and continued to keep increase for two more periods (i.e., four years in total; lag 1, 2), in comparison with the group of adolescents that did not experience a divorce. Over the next measurement, the difference between the adolescents who did and did not experience a divorce remained constant, see also Fig. 1.

**Table 2:** Longitudinal effects of parental post-divorce status on emotional and behavioral problems (*N* = 2230)

Fixed effects	YSR externalizing problems		YSR internalizing problems	
	Coefficient (SE)	*p*	Coefficient (SE)	*p*
Intercept	0.21 (0.01)	<0.001	0.27 (0.01)	<0.001
Time period^a^				
T1	0.02 (0.01)	0.002	0.11 (0.01)	<0.001
T2	0.06 (0.01)	<0.001	0.11 (0.01)	<0.001
T3	0.08 (0.01)	0.001	0.10 (0.01)	<0.001
T4	Ref.	−	−	−
Male (vs. female)	0.01 (0.01)	0.579	− 0.09 (0.01)	<0.001
Time period^a^ by gender				
T1*male	0.06 (0.01)	<0.001	0.04 (0.01)	0.002
T2*male	0.01 (0.01)	0.195	- 0.04 (0.01)	0.007
T3*male	0.02 (0.01)	0.051	- 0.06 (0.01)	<0.001
T4*male	Ref.	−	−	−
Time period^a^ in relation to parental divorce				
Post-divorce status^a^	0.03 (0.01)	0.009	0.03 (0.01)	0.037
First period after post-divorce status^b^ (Lag1)	0.06 (0.01)	<0.001	0.05 (0.01)	0.002
Second period after post-divorce status^b^ (Lag2)	0.06 (0.01)	<0.001	0.07 (0.02)	<0.001
Third period after post-divorce status^b^ (Lag3)	0.07 (0.02)	<0.001	0.08 (0.02)	<0.001
Time period^a^ in relation to parental divorce by gender				
Post-divorce status^b^*male	0.01 (0.02)	0.590	− 0.00 (0.02)	0.741
First period after post-divorce status^b^ (Lag1)*male	0.00 (0.02)	0.911	− 0.02 (0.02)	0.410
Second period after post-divorce status^b^ (Lag2)*male	0.01 (0.02)	0.520	− 0.05 (0.03)	0.045
Third period after post-divorce^b^ (Lag3)*male	0.03 (0.02)	0.153	− 0.00 (0.03)	0.926
Random effects				
Random intercept variance	0.017 (0.000)		0.025 (0.001)	
Residual variance	0.023 (0.001)		0.031 (0.001)	

^a^T1 in 2001/2002, T2 in 2003/2004, T3 in 2006/2007, T4 in 2009/2010

^b^*Post-divorce status T1-4* Parental divorce during period since previous measurement, *SE* standard error, *Ref.* reference category, *T1-4* measurement T1–T4

Furthermore, males and females had different internalizing problem trends over time with males experiencing significantly less internalizing problems. However, parental divorce did not have a significantly different effect for males compared with females for externalizing problems.

Table 3 shows the results of the multilevel analysis comparing the effect of pre-divorce status and post-divorce status on externalizing and internalizing problems. In addition to Table 2, the analysis showed that pre-divorce status did not show a significant effect on either externalizing or internalizing problems.

**Table 3:** Longitudinal effects of parental pre- and post-divorce status on emotional and behavioral problems (*N* = 2230)

Fixed effects	YSR externalizing problems		YSR internalizing problems	
	Coefficient (SE)	*p*	Coefficient (SE)	*P*
Intercept	0.28 (0.01)	<0.001	0.37 (0.01)	<0.001
Time period^a^				
T1	− 0.05 (0.01)	<0.001	0.01 (0.01)	0.123
T2	− 0.02 (0.01)	0.004	0.01 (0.01)	0.220
T3	Ref.	**–**	**–**	**–**
Male (vs. female)	0.03 (0.01)	0.014	− 0.15 (0.01)	<0.001
Time period^a^ by gender				
T1*male	0.05 (0.01)	<0.001	0.10 (0.01)	<0.001
T2*male	0.00 (0.01)	0.102	− 0.04 (0.01)	0.007
T3*male	Ref.	**–**	**–**	**–**
Time period^a^ in relation to parental divorce				
Pre-divorce status^c^	0.02 (0.02)	0.200	**− **0.02 (0.02)	0.272
Pre-divorce status^c^*male	**− **0.02 (0.03)	0.405	**− **0.01 (0.03)	0.786
Post-divorce status^b^	0.04 (0.01)	0.002	0.02 (0.01)	0.239
First period after post-divorce status^b^ (Lag1)	0.07 (0.01)	<0.001	0.03 (0.02)	0.048
Second period after post-divorce status^b^ (Lag2)	0.07 (0.02)	<0.001	0.05 (0.02)	0.010
Time period^a^ in relation to parental divorce^b^ by gender				
Post-divorce status*male	0.00 (0.02)	0.997	**− **0.00 (0.02)	0.955
First period after post-divorce status^b^ (Lag1)*male	**− **0.01 (0.02)	0.629	**− **0.01 (0.02)	0.600
Second period after post-divorce status^b^ (Lag2)*male	0.02 (0.02)	0.452	**− **0.03 (0.032)	0.251
Random effects				
Random intercept variance	0.017 (0.001)		0.026 (0.001)	
Residual variance	0.022 (0.001)		0.029 (0.001)	

^a^T1 in 2001/2002, T2 in 2003/2004, T3 in 2006/2007, T4 in 2009/2010

^b^*Post-divorce status T1-4* parental divorce during period since previous measurement

^c^*Pre-divorce status* period before parental divorce was measured, *SE* standard error, *Ref.* reference category, *T1-4* measurement T1-T4

## Discussion

We found that levels of EBP in adolescents were significantly increased post-parental divorce and that this increase persisted and even enlarged over time. No increase was found before divorce. The effects of a parental divorce on both behavioral and emotional problems never diminish and even increase in the first periods when comparing with adolescents who did not experience a parental divorce.

### Longitudinal effects of parental divorce on emotional and behavioral problems

Parental divorce during adolescence increased adolescent’s risk to develop both emotional and behavioral problems, and this effect increased even further for an additional four years. With a standard error of around 0.2 (this is approximately the SD for internalizing and externalizing continuous scores at wave 1), sizes of the effect of divorce on EBP ranged between 0.15 and 0.35 which could be interpreted as small effects [19]. These findings are in line with previous studies that have identified adolescents as specifically vulnerable to stressful events such as parental divorce [2, 4] due to the high amount of physical, mental, emotional, and social changes during this developmental phase laying the foundation for future health patterns [20, 21]. It has to be noted that especially adolescents who have experienced parental divorce at a very young age (before the age of 10–12) are frequently exposed to socioeconomic effects of divorce such as living in a smaller home, in a precarious neighborhood, attending a school with lower academic performance during a substantial period of time. These living conditions may lead to higher risks of, e.g., substance use, emotional and behavioral problems, poorer school attainment, and delinquency compared to the adolescents who have experienced a post-divorce status at later waves, as they have lasted longer. This may result in more extreme increases of emotional and behavioral problems [1]. Furthermore, the increase in emotional behavioral problems over time found in this study may also be explained by the exposure duration to poorer living conditions (with unmet daily living needs) after parental divorce, that of increasing adolescent's age, and that of other possible problems well-known to be associated with lower SES and increasing age such as substance use and school difficulties. To examine a potential different effect of parental divorce before T1, a sensitivity analysis was performed including an interaction effect of T1 and post-divorce status at T1 to differentiate between the effects of post-divorce status at T1 and a post-divorce status at a later wave. We did this by including the interaction effect of T1 with post-divorce status and the long-term post-divorce effects (lag variables). This sensitivity analysis showed no statistically significant differences in the effects on EBP for a post-divorce status before T1 and at a later wave.

SES was considered an important factor in this study, but data on SES were only collected at baseline (T1) and not at other waves, limiting the possibility to study the causal effects via SES on the studied outcome variables. We have performed a sensitivity analysis adjusting for maternal educational level to capture a full potential confounding effect, a variable that is not thought to change due to divorce and influences both SES (even before children are born) and the risk of divorce [22]. This sensitivity analysis did not show any different results, indicating that the education level of the mother did not influence the relationship between divorce and EBP.

Recent studies found that adolescence is the time of fundamental restructuring of the brain; its heightened plasticity makes adolescents more vulnerable to environmental factors, such as parental divorce, which can have major, lasting effects on cortical circuitry and thus on long-term behavioral responses [23, 24]. Our study confirms in a large sample that parental divorce during adolescence indeed has significant long-term effects for individual’s mental health.

Our findings further showed that effects of parental divorce on emotional problems were larger for girls than boys, two years after the event of parental divorce, but did not differ by gender for behavioral problems. This finding is in line with previous research that consistently shows that males display less internalizing problems than females after parental divorce [14, 15]. The gender differences found in our study imply that the effects of parental divorce may become visible in a very different manner in girls and boys which has to be taken into account in the development and offering of psychological support and therapeutic programs.

### Timing of occurrence of emotional and behavioral problems relative to parental divorce

We found that both emotional and behavioral problems occur more in adolescents during a post-divorce status than a pre-divorce showing the importance of family circumstances after the divorce [1]. These findings are in line with previous research that confirm that adolescents of divorce experience heightened emotional problems after their parent’s divorce [14, 15]. Our findings highlight the negative effects of parental divorce on the psychological health and well-being of adolescents and the urgency to recognize the period after a parental divorce as a critical period for offspring’s development and mental health. This suggests that the actual family disruption and changes in life circumstances after the divorce (e.g., financial situation, relocation, estrangement from one parent, and feelings of guilt) may be more influential on the adolescent’s emotional and behavioral problems than pre-divorce conflicts [25]. Increased attention thus needs to be paid to adolescents whose parents have recently divorced and provide them with the necessary support and resources to make the transition to the new family and life structures as easy as possible.

### Strengths and limitations

This study has several strengths. First, it made use of a large longitudinal population cohort study, TRAILS, that benefits from high retention rates and representativeness of the general population [26]. Second, our use of multilevel analyses allowed to analyze longitudinal nested data in the best way [27] and to analyze subjects one by one in terms of multiple repeated observations that are nested within the individuals. Lastly, this study benefits from multiple measurement waves making it possible to look at changes over time.

Our study also has some limitations. First, due to the nature of the longitudinal data we used, loss to follow-up needs to be considered. Despite a relatively high retention rate, the loss of participants may have led to an effect underestimation as the lost participants were mostly male individuals with low parental socioeconomic status, already divorced parents, and overall lower EBP scores in the waves before drop-out [17, 26]. Second, due to measurement limitations of the divorce status, such as varying time laps between actual moment of divorce and measurement (about 11 years of age) and a lack of data for a pre-divorce status before T1, the moment of divorce could not be measured fully accurately. This timing measurement error does not affect the relationship pre- versus post-divorce status with EBP, and their long-term effects. Yearly measures would be more accurate but have not been feasible within this large cohort study. Thirdly, the variables pre-divorce and post-divorce probably may not have fully covered (the duration of) conflict behaviors between parents prior and subsequent to divorce, leading to some underestimation of the effect of a pre-divorce status (reflecting conflicts between parents) on the adolescent’s mental health.

Due to likely persistent conflicts after the event of a parental divorce, disentangling the effects of conflicts prior and subsequent to the divorce has not been possible in the current study but it is recommended for future research.

### Implications for practice, research, and policy

Our study showed that the actual divorce itself (i.e., the post-divorce status) was significantly associated with the development of both short-term and long-term mental health problems in comparison with the level of problems in adolescents who did not experience a divorce. Considering the recent and rapid increase of (parental) divorces in Europe, it is pivotal to make mental health a main target for future preventive interventions in adolescents experiencing parental divorce.

Further studies are needed to confirm our results, preferably with a refinement of the assessment of parental divorce in multiple measurement waves and a further specification of the pre-divorce status. Further specification of conflict behaviors in the family which may, but not always, lead to divorce are needed to confirm if conflict (potentially leading to a parental divorce) indeed does not have a significant influence on adolescent’s mental health. It is further recommended to investigate the influence of changes of the families’ SES (preferably also for mother and father separately) as a mediating factor between post-divorce status and EBP to accurately disentangle the effects of the event of parental divorce and its consequences for changes in living situation, financial circumstances, and community environment. By integrating interdisciplinary approaches, future studies and intervention programs may considerably contribute to our understanding of the etiology of adolescent mental health and thus to an evidence-based promotion of healthy lives within future generations.

Finally, our findings confirm that policy makers and youth and family services need to pay close attention to divorcing and conflict-laden families with adolescent children to ensure their well-being at any age.

## Conclusions

In conclusion, we found that the phase after the parental divorce, rather than before the divorce, is crucial for the healthy development of adolescents. These effects are long-lasting and highlight the need for better care for children with divorcing parents which may highly contribute to a healthy future.
